# Proteomics Study on Nonallergic Hypersensitivity Induced by Compound 4880 and Ovalbumin

**DOI:** 10.1371/journal.pone.0148262

**Published:** 2016-02-01

**Authors:** Yubin Xu, Na Guo, Deqiang Dou, Xiaoku Ran, Xiande Ma, Haixue Kuang

**Affiliations:** 1 College of Pharmacy, Liaoning University of Traditional Chinese Medicine, Dalian, Liaoning, China; 2 College of pharmacy, Heilongjiang University of Chinese Medicine, Harbin, Heilongjiang, China; 3 Experimental Research Center, China Academy of Chinese Medical Sciences, Beijing, China; University of North Carolina at Chapel Hill, UNITED STATES

## Abstract

Nonallergic hypersensitivity reaction (NHR) accounts for more than 77% of all immune-mediated immediate hypersensitivity reactions and has become a serious threat to public health. Here, proteomics was used to study the NHR mechanism of two typical substances, the compound 4880 and ovalbumin. Twelve different proteins were suggested as potential biomarkers for examining the NHR mechanism, and our results revealed that the mechanism mainly encompassed 2 processes, i.e., generation and effect processes. The generation process could be classified as direct stimulation, complement (classical and alternative), coagulation, kallikrein-kinin, and integrated pathways. Thus glutathione peroxidase 1, terminal complement complex (complement factor 4d and Bb), coagulation 13, kininogen-1, and IgE could be used as candidate biomarkers for the indication of the corresponding pathways respectively, the proteins were further confirmed by ELISA. And the effect process was mainly composed of histamine as well as proteins such as DCD and MYLPF, which could be used as important indices for the symptoms of NHR. Our study differs from previous studies in that C4880 was found to not only be involved in the direct stimulation pathway, but also in the activated complement and kallikrein-kinin pathways through the coagulation pathway. We also report for the first time that ovalbumin-induced NHR could be a combination of the coagulation, classical complement, and integrated pathways.

## Introduction

Nonallergic hypersensitivity (pseudoallergy or idiosyncratic) is a nonimmune hypersensitivity reaction that mimics allergic reactions. The first "anaphylactoid" phenomenon was discovered in 1920 when Karsner [1] intravenously injected colloidal substances in humans and induced anaphylaxis-like symptoms. The typical anaphylactoid reaction was confirmed in the 1990s after intravenous administration of an oil adjuvant vaccine to cattle, and further research indicated that it was caused by its auxiliary Tween-80 and not initiated or mediated by pre-existing IgE antibodies [2]. Subsequently, a series of substances including radiologic contrast agents, non-steroidal anti-inflammatory drugs, analgesics, liposomes, micelles, and vitamin K injection were found to produce anaphylactoid reactions [3–5]. According to revised terminology from 2003, the European Academy of Allergy and Clinical Immunology has suggested that each condition should be categorized as allergic or nonallergic, and terms that are no longer in use are idiosyncrasy (now hypersensitivity), pseudoallergy (now nonallergic hypersensitivity), and anaphylactoid reaction (now nonallergic anaphylaxis). Nonallergic hypersensitivity reaction (NHR) is generally recognized as occurring after the first exposure to antigen and not mediated by pre-existing IgE antibodies, and accounts for more than 77% of all immune-mediated immediate hypersensitivity reactions [6]. The mechanism underlying NHRs has been investigated and 3 pathways, encompassing mast cells directly stimulated by antigens [7], activation of the coagulation sequence [8], and the complement pathway [9], have been proposed. However, most of these studies were primarily focused on the effector substances such as histamine and tryptase [5, 10, 11], and the underlying mechanism is still not completely clear. It is generally known that blood proteins are involved in NHRs; thus, proteomics could be more conducive to revealing the mechanism of NHRs.

Ovalbumin (OVA) has commonly been used as a positive control for type I anaphylactic reactions and can also induce NHRs [12], but its mechanism of action has not been studied. Compound 4880 (C4880) is well recognized for its ability to induce mast cell-dependent, nonspecific anaphylactoid reactions [7]. In addition, due to their susceptibility, brown Norway (BN) rats have been selected as an ideal animal for the evaluation of NHRs [13]. Thus, the NHR mechanisms of BN rats induced by C4880 or OVA were studied for the first time by comprehensive application of proteomics. The objective of the work presented here was to address the following problems: (1) identification of different blood proteins related to NHRs, (2) the differences in the NHR mechanisms between C4880 and OVA, and (3) the exploration of potential biomarkers for mechanistic analysis of NHR-inducing substances.

## Materials and Methods

### Reagents and Materials

The assay kit for histamine was purchased from USCN Life Science Inc. (Wuhan, China). The assay kits for immunoglobulin E (IgE), glutathione peroxidase 1 (Gpx1), coagulation factor 13 (F13), kininogen-1 (Kng1), complement factor Bb (Bb), complement factor C4d (C4d), and terminal complement complex (Sc5b9) were purchased from Nanjing Jiancheng Bioengineering Institute (Nanjing, China). C4880 and OVA were obtained from Sigma-Aldrich (St. Louis, MO, USA). Ultrapure water was prepared with a Milli-Q water purification system (Millipore, France).

### Animal Experiments and Sample Collection

Male BN rats, 200 ± 20 g in weight, were purchased from WeiTongLiHua Co. (Beijing, China), laboratory animal license SCXK (Army): 2012–0001. The animals were kept under SPF laboratory conditions (Center for Animal Experiments of Liaoning University of Traditional Chinese Medicine, Shenyang, China) and received a standard laboratory diet and filtered tap water ad libitum, and cared daily by animal resource Centre. All animal procedures were approved by Liaoning Provincial Animal Welfare and Care Guidelines in accordance with the National Institute of Health’s guidelines regarding the principles of animal care (2004). The experimental animals were housed under the above conditions for a 1-week acclimation period. Special care was taken to minimize animal discomfort during all procedures. The condition of the experimental rats was monitored every one minutes after injection. At the end of the treatment rats were sacrificed by cervical dislocation under general anesthesia induced by hydrate anesthesia. For the one animal that died during the study, pentobarbital was used to anesthetize before it died.

According to the results of preliminary experiments, BN rats were randomly divided into 6 groups, 8 rats per group: control groups (Con_1 and Con_2, saline), C4880 groups (C4880_1 and C4880_2, 10 mg/kg), OVA groups (OVA_1 and OVA_2, 400 mg/kg). Con_2, C4880_2, and OVA_2 were biological replicates. Subsequently, the corresponding test substances (filtered through 0.22 μm pore size sterile filters) were administered intravenously into the BN rats of each group and controls received the same volume of saline; the injection was completed in 20 s. Anaphylactic symptoms were observed in the BN rats 30 min after injection. Symptoms were evaluated by using a scoring system described by Li et al. [14] and the immune toxicity of traditional Chinese medicine, natural medicine (allergic, light allergic reaction) technology guidelines for research as follows: 0, no abnormal reaction; 1, trembling, scratching, and rubbing around the nose; 2, sneezing, coughing, puffiness around the eyes and the mouth, shortness of breath; 3, dyspnea, wheezing, unsteady gait, cyanosis around the mouth and the tail, myasthenia of limbs, convulsions, spasm, rotation, tidal breathing; 4, death.

Thirty minutes after injection, the animals were anesthetized by pentobarbital and blood samples were obtained and collected in chilled tubes containing EDTA-K2 from abdominal aorta, and then centrifuged at 3000 × *g* (10 min, 4°C) to obtain plasma. Aliquots (1.5 mL) of plasma from each rat were collected separately in tubes for proteomics analysis, and then all aliquots were stored at −80°C until analysis.

### Proteomics

#### Protein preparation

To reduce the complexity of samples, the highly abundant proteins were depleted using ProteoMinerTM Kits (Bio-Rad Laboratories, Hercules, CA, USA) according to the manufacturer’s protocol. Samples were eluted in Lysis buffer (7 M Urea, 2 M Thiourea, 4% CHAPS, 40 mM Tris-HCl, pH 8.5) and reduced with 10 mM DTT (final concentration) at 56°C for 1h, followed by alkylation with 55 mM IAM (final concentration) in the darkroom for 1 h. The reduced and alkylated protein mixtures were precipitated by adding 4 × volume of chilled acetone at -20°C overnight. After centrifugation at 4°C, 30,000 × g, the pellet was dissolved in 0.5 M TEAB (Applied Biosystems, Milan, Italy) and sonicated in ice. After centrifuging at 30,000 × *g* at 4°C, an aliquot of the supernatant was taken for determination of protein concentration and purity by Bradford method and SDS-PAGE. The proteins in the supernatant were kept at -80°C for further analysis. (The results are shown in S1 and S2 Tables, and S1 Fig)

The protein preparation was performed as previously described with minor modification [15, 16]. Briefly, the plasma samples were crushed into powder in liquid nitrogen and extracted with lysis buffer (7 M urea, 2 M thiourea, 4% CHAPS, 40 mM Tris-HCl, pH 8.5) containing 1 mM PMSF and 2 mM EDTA (final concentrations). Then, 10 mM DTT (final concentration) was added to the samples for 5 min and the resulting suspension was sonicated at 200 W for 15 min, and then centrifuged at 30,000 × *g* (4°C, 15 min) to provide the supernatant, which was mixed with 5 volumes of chilled acetone containing 10% (v/v) trichloroacetic acid and incubated at −20°C overnight. After centrifugation, the supernatant was discarded and chilled acetone was used to wash the precipitate three times. Then, the resulting air-dried pellets were dissolved in lysis buffer (7 M urea, 2 M thiourea, 4% NP40, 20 mM Tris-HCl, pH 8.0–8.5) and sonicated, centrifuged, and then 10 mM DTT (final concentration) was added to reduce the proteins at 56°C for 1 h. Subsequently, the cysteines were blocked by 55 mM iodoacetamide (final concentration) for 1 h in total darkness. The proteins were precipitated with 5 volumes of chilled acetone at −20°C for 2 h. After centrifugation at 30,000 × *g* and 4°C, the supernatants were discarded and 500 μL 0.5 M tetraethylammonium bromide (Applied Biosystems, Milan, Italy) was used to dissolve the air-dried pellets, which were sonicated for 15 min at 200 W. Finally, samples were centrifuged at 30,000 × *g* (4°C, 15 min). The supernatant was quantified using the Bradford method. The proteins in the supernatant were stored at −80°C for further analysis.

#### iTRAQ labeling and SCX fractionation

The iTRAQ assay was the same as that used in a previous study [17]. Briefly, samples were labeled with the iTRAQ tags as follows: Sample Con_1 (113 tag), Sample C4880_1 (117 tag), Sample OVA_1 (121 tag), Sample Con_2 (115 tag), Sample C4880_2 (119 tag), and Sample OVA_2 (114 tag). The labeled peptide mixtures were incubated for 2 h at room temperature, and then pooled and dried by vacuum centrifugation.

SCX chromatography with an LC-20AB HPLC Pump system (Shimadzu, Kyoto, Japan) was used to purify and separate the peptide mixtures. The iTRAQ-labeled peptide mixtures were dissolved in 4 mL buffer A (25 mM NaH_2_PO_4_ in 25% acetonitrile (ACN), pH 2.7) and loaded onto a 4.6 × 250 mm Ultremex SCX column containing 5-μm particles (Phenomenex, Torrance, CA, USA). The peptides were eluted at a flow rate of 1 mL/min using buffers A and B (25 mM NaH_2_PO_4_, 1 M KCl in 25% ACN, pH 2.7). The gradient program was as follows: 0–10 min, 0% B; 10–37 min, 5% B to 60% B; 37–38 min, 60% B to 100% B; 38–39 min, 100% B; 39–40 min, 100% B to 0% B; 40–50 min, 0% B. The fractions were collected every 1 min while measuring the absorbance at 214 nm. A Strata X C18 column (Phenomenex) was used to desalt the 20 fractions, which were then dried under vacuum.

#### LC-ESI-MS/MS analysis based on Triple TOF 5600

The LC-ESI-MS/MS analysis was conducted as previously described [17] with minor modification. Briefly, buffer A (5% ACN, 0.1% formic acid) was used to dissolve each fraction, and the fractions were centrifuged at 20,000 × *g* (10 min): 0.5 μg/μL on average was the final concentration of peptide. Supernatant (10 μL) was loaded on an LC-20AD nanoHPLC (Shimadzu) and the peptides were eluted at 300 nL/min using buffer A and B (95% ACN, 0.1% formic acid). The gradient program was as follows: 0–35 min, 2% B to 35% B; 35–40 min, 35% B to 60% B; 40–42 min, 60% B to 80% B; 42–46 min, 80% B; 46–47 min, 80% B to 5% B.

The TripleTOF 5600 System (AB SCIEX, Concord, Canada) fitted with a Nanospray III source (AB SCIEX) and a pulled quartz tip as the emitter (New Objectives, Woburn, MA, USA) was used to acquire data and the TF 1.6 Analyst was used to control the AB Sciex 5600. The conditions were as follows: ion spray voltage, 2.5 kV; curtain gas, 30 psi; nebulizer gas, 15 psi; interface heater temperature, 150°C; TOF MS scans, greater than 30,000 FWHM; for IDA, survey scans, 250 ms (30 product ion scans were collected if exceeding a threshold of 120 counts/s) with a 2+ to 5+ charge-state; total cycle time, 3.3 s; Q2 transmission window, 100 Da for 100%; pulser frequency value, 11 kHz; sweeping collision energy, 35 ± 5 eV; dynamic exclusion, 1/2 of peak width (15 s); and then the precursor was refreshed off the exclusion list.

#### Data analysis

The Mascot search engine (Matrix Science, London, UK; version 2.3.02) was used to query against Uniprot_Rat database containing 36508 sequences (http://www.uniprot.org/uniprot/?query=taxonomy%3ARattus&sort=score, 2015-11-18) to identify proteins.

For protein identification, the parameters of Mascot were set as follows: intact peptide mass tolerance, 0.05 Da; fragmented ions, 0.1 Da; trypsin digests, allowance for one missed cleavage; potential variable modifications, Gln -> pyro-Glu (N-term Q), Oxidation (M), Deamidated (NQ); fixed modifications, Carbamidomethyl (C), iTRAQ8plex (N-term), iTRAQ8plex (K); charge states of peptides, +2 and +3. Specifically, an automatic decoy database search was performed in Mascot by choosing the decoy checkbox in which a random sequence database is generated and tested for raw spectra as well as the real database. At least one unique peptide had to be identified with 95% confidence to reduce the probability of false peptides.

For protein quantitation, it was required that a protein contains at least two unique peptides. The p-value and quantitative protein ratios were weighted and normalized by the median ratio in Mascot. Ratios with p-values < 0.05 and fold changes > 1.2 were considered significant.

The Blast2GO program was run against the non-redundant protein database (NCBI) to conduct the functional annotations of the proteins. The KEGG database (http://www.genome.jp/kegg/) and the COG database (http://www.ncbi.nlm.nih.gov/COG/) were used to classify and group these identified proteins.

### ELISA assay

BN rats were randomly divided into the following 3 groups with 10 rats per group: control, C4880, and OVA. The experimental procedure was identical to that used in section (Animal Experiments and Sample Collection), and the plasma of each rat was collected into tubes and stored at −80°C until analysis. Histamine, IgE, Gpx1, F13, Kng1, Bb, C4d, and Sc5b9 levels were then determined by using ELISA kits following the manufacturers’ instructions.

### Statistical analysis

All values are expressed as means ± SD. The significance of differences among the means of the C4880, OVA, and control groups was compared by one-way ANOVA followed by Dunnett method using the Statistical Package for Social Science program (SPSS 20.0, Chicago, IL, USA). The significance threshold was set at p < 0.05 for this test.

## Results

### Animal behavior study

The symptoms of BN rats in control groups were regarded as normal. Those of BN rats in the C4880 group showed dyspnea, unsteady gait, myasthenia of limbs, convulsions, spasm, and death. The BN rats in the OVA group exhibited trembling, scratching of the nose, and shortness of breath. The scores for each group are shown in Table 1, and the results indicate that the rats in the OVA and C4880 groups exhibited serious NHR.

**Table 1:** The symptom scores of BN rats.

group	Dosage (mg/kg)	Judgment of the typical symptom score /count					Symptom scores
		0	1	2	3	4	
Control	-	10	0	0	0	0	0
C4880	10	0	0	0	9	1	3.1±0.32
OVA	400	0	7	3	0	0	1.3±0.48

### Proteomics study

#### Rat plasma proteome and its functional categories

**Fig 1:**
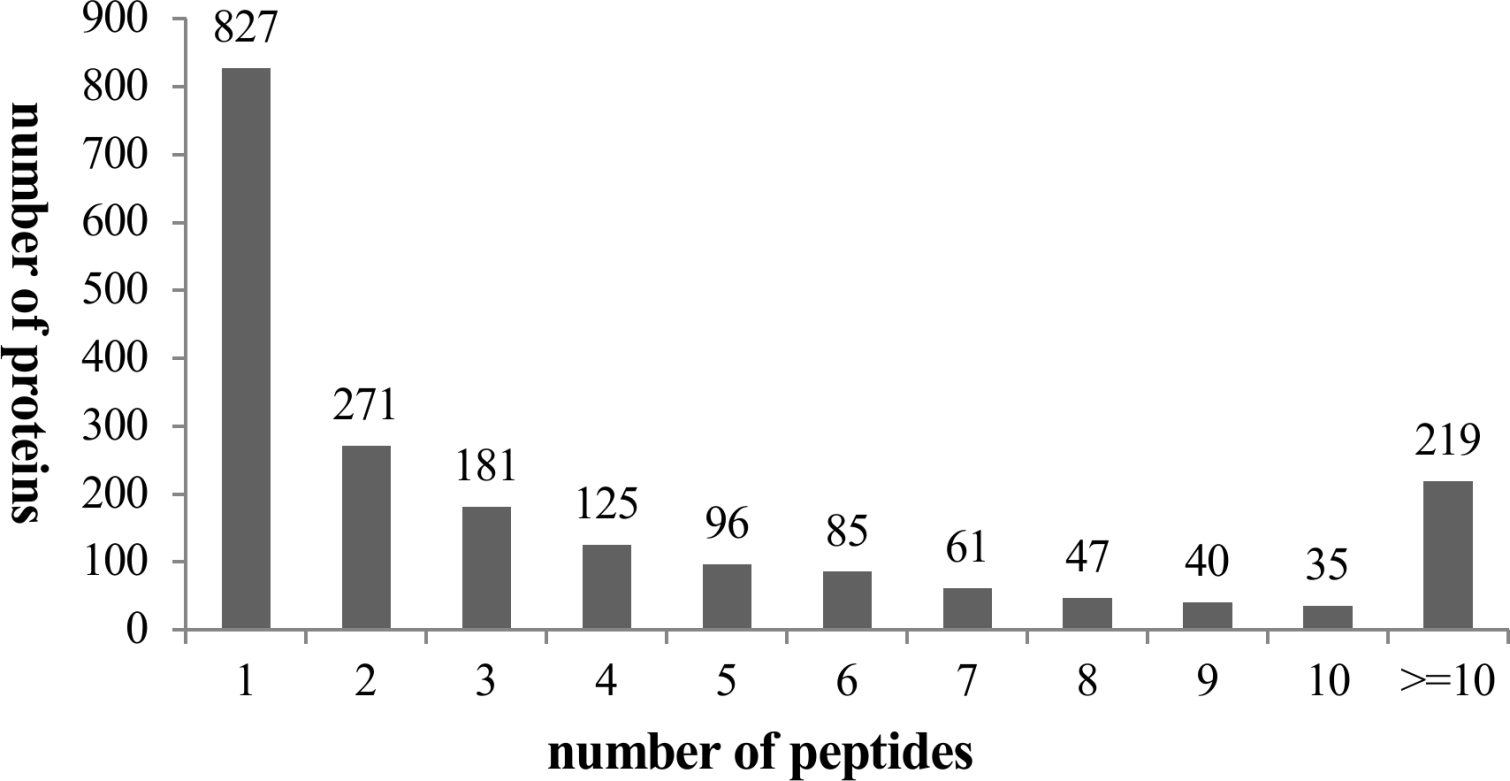
The number of distinct peptides identified in a protein. The peptides were identified using Mascot, based on a 95% confidence level.

Information from Con_1, C4880_1 and OVA_1 and their biological replicates (Con_2, C4880_2 and OVA_2) were analyzed using Mascot. The total number of distinct peptides identified by Mascot was 10585, yielding 1987 proteins (S3 Table). The false discovery rate of protein identification obtained by Mascot is 1.6%. Fig 1 shows the number of peptides identified in a protein. These results clearly show that 40–42% of the proteins were identified by a single peptide, more than 50% of the proteins were identified by at least two distinct peptides, and approximately 11% of the proteins were identified by more than eleven peptides and these were proteins in higher abundance.

**Fig 2:**
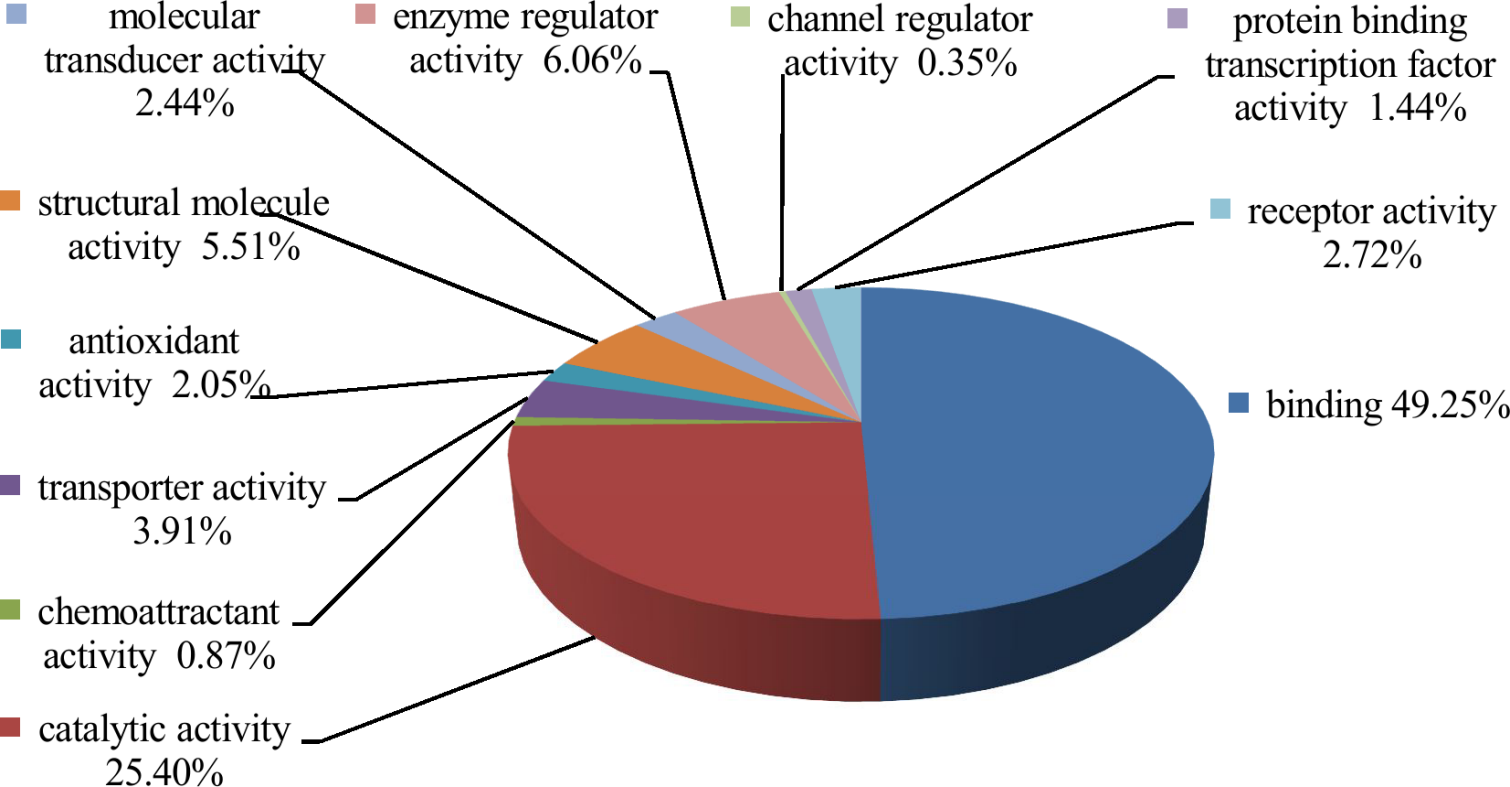
Molecular functions of the total proteins.

Fig 2 shows the functional categories of the 1987 proteins identified in this study. The proteins that were involved in binding and catalytic activity were 49.25% and 25.40%, respectively, due to metabolism occurring in the plasma. The remaining proteins included regulatory enzymes, structural molecules, transporters, receptors, molecular transducers, antioxidants, protein-binding transcription factors, chemoattractants, and regulators of channel activity.

#### Differentially expressed proteins

**Fig 3:**
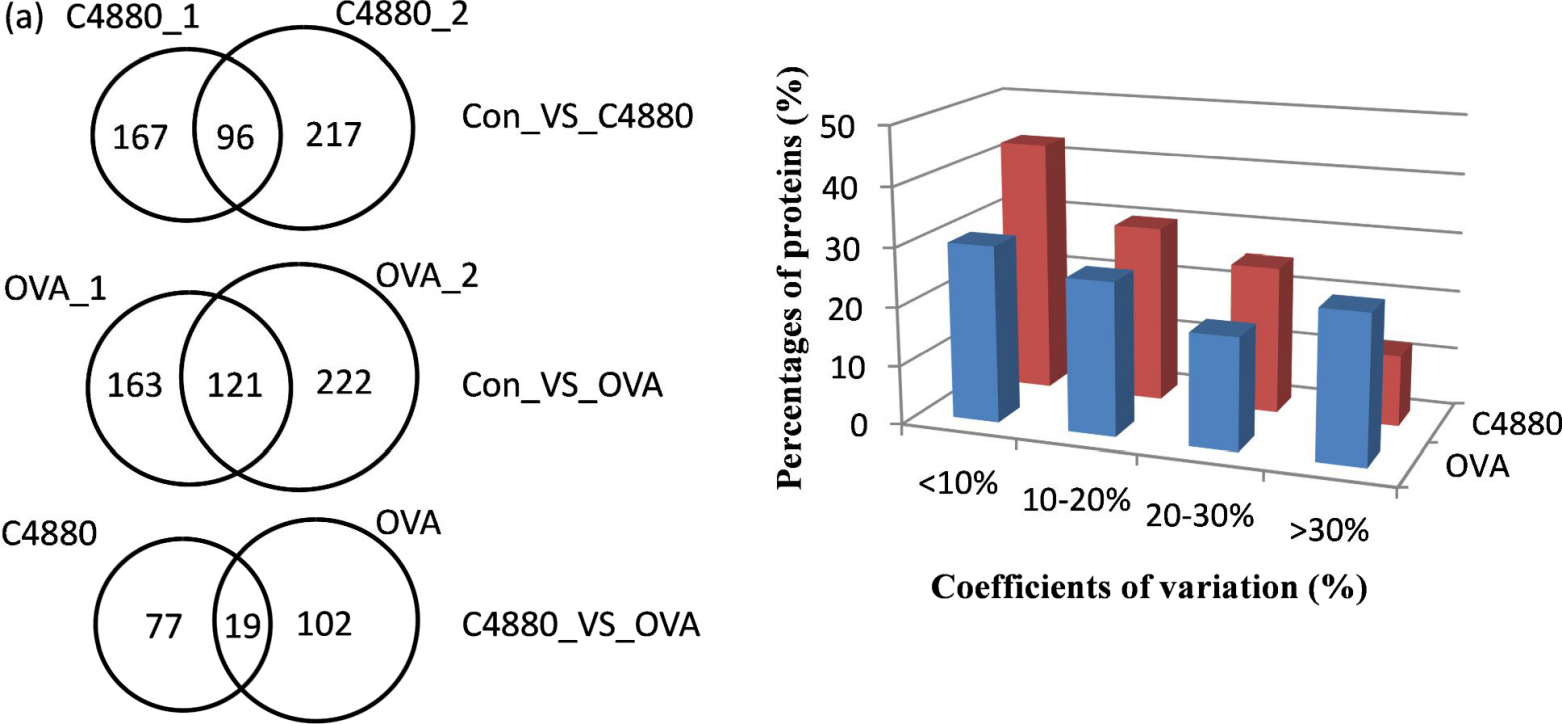
Reproducibility of protein identification. (a) Biologically replicate experiment was processed with iTRAQ analyzing procedure: there were 96 differentially expressed proteins identified in the C4880 group and 121 differential proteins identified in OVA group; the number of total differential proteins in C4880 and OVA groups was 198, 19 differential proteins were common. (b) Coefficients of variation of C4880 and OVA groups comparing with control group.

Only the proteins identified in experiment 1 (Con_1, C4880_1, and OVA_1 groups) and the biological replicate experiment 2 (Con_2, C4880_2, and OVA_2 groups) with ratios showing p < 0.05 and fold changes > 1.2 were considered differentially expressed proteins. Comparing the 167 proteins identified in Con_1_VS_C4880_1 with the 217 proteins identified in the biological replicate (Con_2_VS_C4880_2), there were 96 differentially expressed proteins identified in the C4880 group. Comparing the 163 proteins identified in Con_1_VS_OVA_1 with the 222 proteins identified in the biological replicate (Con_2_VS_OVA_2), there were 121 differentially expressed proteins identified in the OVA group; the total number of differentially expressed proteins in the C4880 and OVA groups was 198 (Fig 3A).

In the biological replicate experiment, among the 96 differentially expressed proteins in group C4880, 43%, 30%, and 25% of proteins had coefficients of variation under 10%, 10–20%, and 20–30%, respectively, and 12% of proteins exceeded 30%. Among the 121 differentially expressed proteins in the OVA group, 30%, 26%, and 19% of proteins had coefficients of variation under 10%, 10–20%, and 20–30%, respectively, and 25% of proteins exceeded 30% (Fig 3B).

Among the 198 differentially expressed proteins, 12 which related to NHR and the generation pathways were further submitted for functional analysis with reference to the COG database (http://www.ncbi.nlm.nih.gov/COG/) and KEGG database (http://www.genome.jp/kegg/). Table 2 shows the UniProt accession number, description, gene name, ratio, KEGG pathway, and functional ontology. Intriguingly, seven of the 12 proteins (F13b, F12, C1qa, Kng1, Serpind1, C2, and C6) are involved in complement and coagulation cascade pathways. F13b and Prdx2 were upregulated in both the C4880 and OVA groups; F12, Cndp1, MYLPF, and DCD were downregulated in both the C4880 and OVA groups; Kng1 was upregulated in the C4880 group; C1qa and Gpx1 were downregulated in the C4880 group; and Serpind1, C2, and C6 were downregulated in the OVA group.

**Table 2:** Proteins identified and quantified with iTRAQ technology detected by LC-ESI-MS/MS.

UniProt accession number	Description	Gene name	Con-VS-OVA	Con-VS-C4880	KEGG pathway	Functional ontology
B1H260	Coagulation factor XIII B chain	F13b	1.253*	2.04*	Complement and coagulation cascades (k03906)	—
D3ZTE0	Coagulation factor XII	F12	0.749*	0.82*	Complement and coagulation cascades (k01328)	misfolded protein binding; serine-type endopeptidase activity; serine-type aminopeptidase activity
P35704	Peroxiredoxin-2	Prdx2	1.204*	1.258*	—	protein binding; thioredoxin peroxidase activity; selenium binding
Q66HG3	Beta-Ala-His dipeptidase	Cndp1	0.47*	0.694*	beta-Alanine metabolism/Arginine and proline metabolism/Histidine metabolism (k05604)	metal ion binding; metallopeptidase activity; dipeptidase activity; tripeptidase activity; carboxypeptidase activity
P02608	Myosin regulatory light chain 2, skeletal muscle isoform type 2	MYLPF	0.705*	0.516*	Leukocyte transendothelial migration/Focal adhesion/Regulation of actin cytoskeleton (k12758)	structural constituent of muscle; calcium ion binding
Q71DI1	Dermcidin	DCD	0.638*	0.7*	—	—
P31720	Complement C1q subcomponent subunit A	C1qa	0.927	0.676*	Complement and coagulation cascades (k03986)	protein binding
Q5PQU1	Kininogen-1	Kng1	0.946	1.92*	Complement and coagulation cascades (K03898)	receptor binding; protease binding; cysteine-type endopeptidase inhibitor activity
P04041	Glutathione peroxidase 1	Gpx1	1.755	0.638*	Arachidonic acid metabolism/ Glutathione metabolism (K00432)	glutathione binding; SH3 domain binding; glutathione peroxidase activity; phospholipid-hydroperoxide glutathione peroxidase activity; selenium binding; endopeptidase inhibitor activity
Q64268	Heparin cofactor 2	Serpind1	0.616*	1.153	Complement and coagulation cascades (K03912)	serine-type endopeptidase inhibitor activity; heparin binding
Q811M5	Complement component C6	C6	0.68*	1.081	Complement and coagulation cascades (K03995)	protein binding
Q6MG73	Complement C2	C2	0.685*	1.121	Complement and coagulation cascades (K01332)	threonine-type endopeptidase activity; lipopolysaccharide binding; protein binding; serine-type endopeptidase activity

Compared to control group

*p < 0.05.

**Fig 4:**
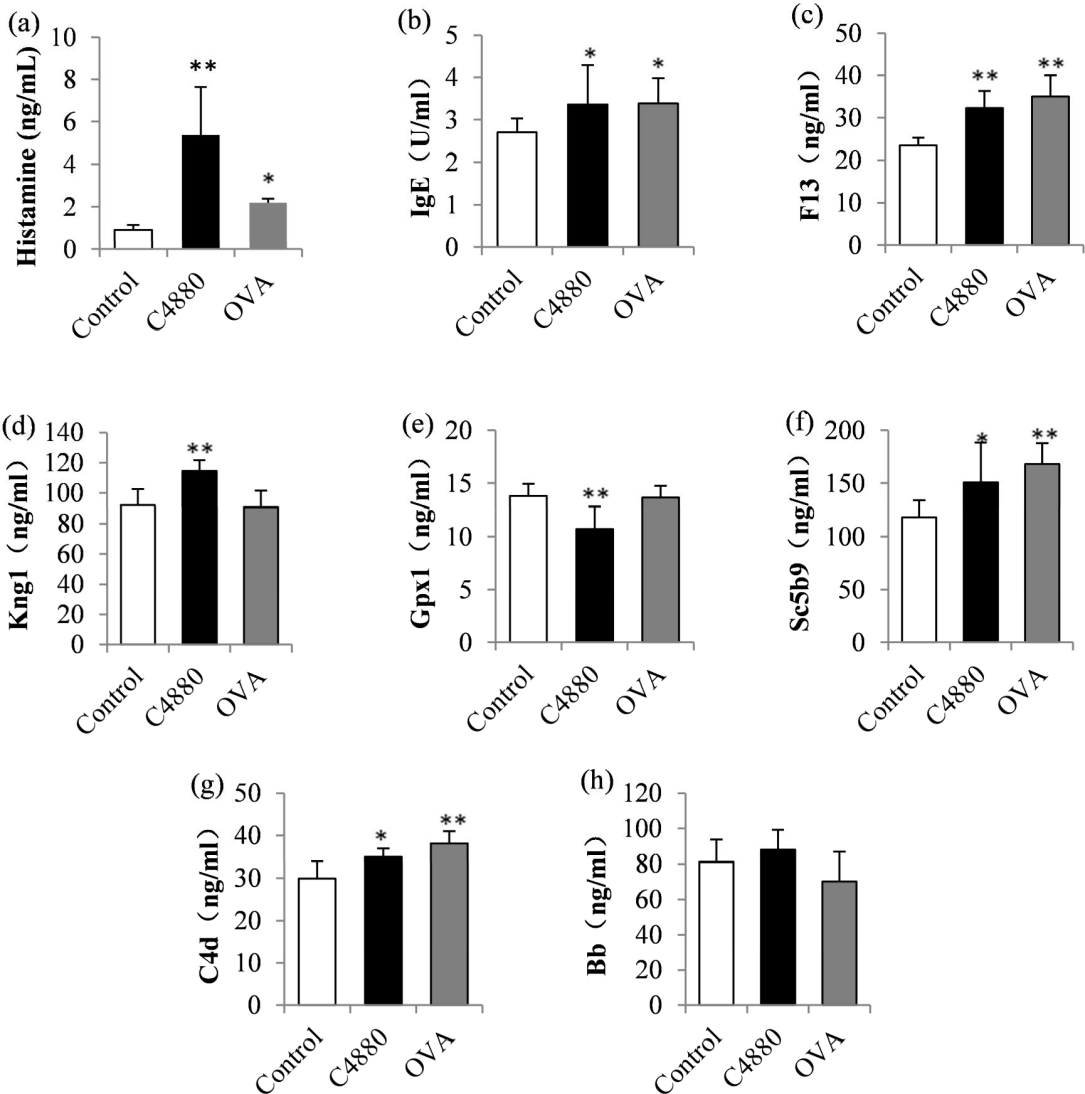
The histamine, IgE, F13, Kng1, Gpx1, Sc5b9, C4d and Bb levels of BN rats by ELISA assay. All values are expressed as means ± SD and analyzed through one-way ANOVA followed by Dunnett method to determine significance between the control and experimental groups. Compared to control group, *P<0.05, **P<0.01.

To confirm the results drawn from proteomics and reveal the NHR action routes of C4880 and OVA, 7 proteins (F13, Kng1, Gpx1, IgE, Sc5b9, C4d, and Bb) regarded as key biomarkers of NHR were selected and assayed by ELISA. Simultaneously, histamine levels were also measured to indicate the NHR degree. The results show that the histamine levels were significantly increased in the C4880 (p < 0.01) and OVA (p < 0.05) groups compared with the control group, indicating the occurrence of NHRs in these BN rats. The IgE levels were significantly higher in C4880 (p < 0.05) and OVA (p < 0.05) groups compared with the control group. The F13 levels were significantly increased in C4880 (p < 0.01) and OVA (p < 0.01) groups compared with the control group. The Kng1 levels were significantly increased and Gpx1 levels were significantly decreased in the C4880 group (p < 0.01) versus the control group. The Sc5b9 and C4d levels were significantly increased in the C4880 (p < 0.05) and OVA (p < 0.01) groups versus the control group. There were no significant differences of Bb levels in both groups compared with the control group (Fig 4).

## Discussion

Acute allergic reactions mainly include type I anaphylaxis and NHR, and the latter is often caused by injections of such things as ginko biloba and taxol; increasing attention has recently been paid to NHR. Previous studies had found that BN rats were more suitable for NHR evaluation. It is generally known that C4880 can induce mast cell-dependent NHR that is commonly attributed to a direct, receptor-bypassing property to activate G proteins to release histamine from mast cells [18]. However, Palomaki and Laitinen [19] concluded that C4880 is not a receptor-bypassing, general G protein activator but rather activates phospholipase D, leading to generation of endogenous lysophosphatidic acid receptor-activating phospholipids, which increases the rate of GTPγS binding to G proteins, resulting in the release of histamine from mast cells [7]. Conversely, McNeil et al. [20] discovered that Mrgprb2, the orthologue of G protein-coupled receptor MRGPRX2, could not globally impair IgE or G protein-coupled receptor-mediated mast cell signaling through studies in Mrgprb2-mutant mice sensitized to OVA, but C4880-induced mast cell activation and tissue histamine release were essentially abolished in Mrgprb2-mutant mast cells. Thus, it can be concluded that C4880-induced NHR is associated with a direct stimulatory pathway, but the biomarker results provided no clear indication of the exact pathway. Therefore, proteomics and metabolomics were used to study the mechanism of NHR and analyze how C4880 and OVA can give rise to NHR, resulting in finding their potential biomarkers for NHR.

**Fig 5:**
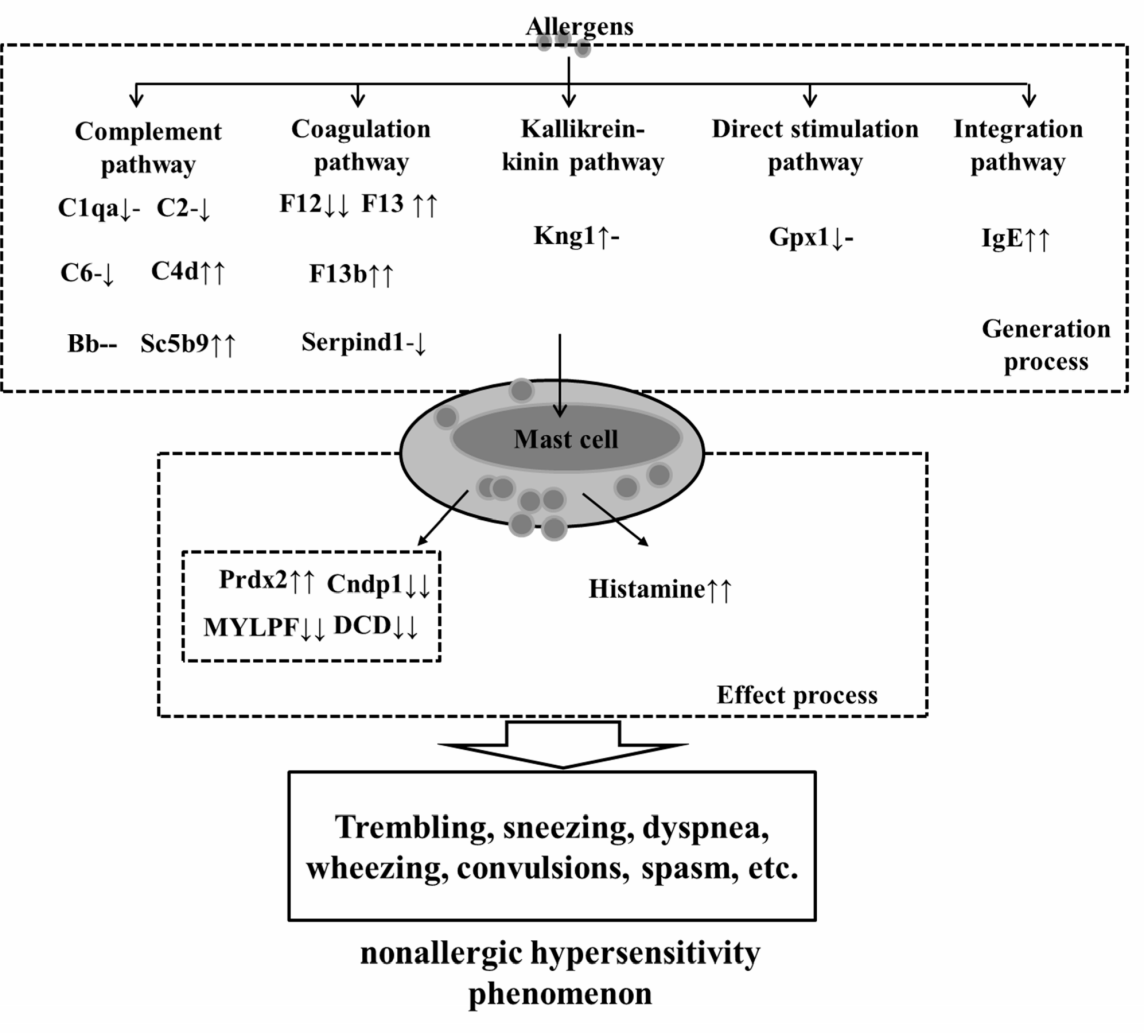
A proposed generation and effect mechanism study of nonallergic hypersensitivity reaction according to KEGG nomenclature through proteomics. The levels of potential biomarkers were labeled with “↑” up-regulated, “↓” down-regulated and “-” no significant difference comparing with control group, the left was C4880 group and the right was OVA group.

According to the KEGG database (http://www.genome.jp/kegg/pathway.html) and some reports [4, 7, 18, 19, 21], the NHR mechanism mainly encompasses 2 processes, i.e. generation and effect, as determined in this study (Fig 5). It can be concluded that the generation process (antigens activating mast cells through signal transduction) can be classified as direct stimulation, complement, coagulation, kallikrein-kinin, and IgE integrated with anaphylatoxin-triggered pathways (S2 Fig); the effect process (activated mast cells releasing effector substances) is mainly composed of small molecules and proteins such as histamine, which is also an important index for the indication of type I hypersensitivity reactions.

### Generation process

IgE has often been used to discriminate type I anaphylaxis and NHR [2], but another study has also shown that allergens can cause NHR through IgE integrated with the anaphylatoxin-triggered pathway (integrated pathway) [3]. A previous study showed that IgE could not be obviously altered when treated with a low dose of C4880, but there was a significant difference in the present study, suggesting that IgE may be correlated with allergen species, and in a dose-dependent manner. Thus, IgE is an important auxiliary index for this pathway.

F12, which is the initial factor of the coagulation cascade pathway [3, 22], was downregulated in the C4880 and OVA groups, while the key factor F13 and its fragment F13b were upregulated. Thus, it can be concluded that prothrombin is activated to further generate F12a from the cleavage of F12, and then F12a and F13 participate in blood coagulation together through a series of cascade reactions [23], suggesting that C4880 and OVA are involved in the coagulation pathway. Serpind1, in inhibitory factor of the coagulation cascade pathway [24], was downregulated in the OVA group but displayed no obvious change in the C4880 group, indicating that the procoagulant effect of OVA is stronger than that of C4880. Thus, F12 and F13 can be used as important indices for the coagulation pathway, while Serpind1 can be used as an auxiliary marker to judge the strength of the procoagulant effect of antigens.

C1qa is a subcomponent of the first component (C1q) of complement C1; binding between C1q and immunoglobulins is required for activation of subsequent complement components [25]. Furthermore, an *in vitro* study also indicated that antigens and some apoptotic cells can also be combined with C1q to trigger complement activation in the absence of antibody [26]. In the present study, C1qa was downregulated in the C4880 group, but no change was observed in the OVA group. The complement components C2 [27] and C6 [28] were downregulated in the OVA group, while Sc5b9 and C4d were upregulated in both groups, and Bb was unchanged in both groups, suggesting that C4880 and OVA are involved in the classical complement pathway [29]. However, a previous *in vitro* study indicated that Sc5b9 showed almost no change after C4880 treatment, suggesting that C4880 may indirectly stimulate the complement system to consume C1q through the coagulation system [30] while OVA directly stimulates the complement system, and the levels of complement components would change with time and antigen type. Taken together, these results suggest that Sc5b9 can be used as a biomarker for the complement pathway, C4d can be used as a biomarker for the classical complement pathway, and Bb can be used as a biomarker for the alternative complement pathway [31].

Kng1, which is cleaved by plasma kallikrein, can lead to the liberation of bradykinin. Sala-Cunill et al. discovered that deficiency in or pharmacological inhibition of plasma kallikrein or Kng1 largely attenuated allergen/IgE-mediated mast cell hyperresponsiveness in mice [32]. Bradykinin is an extremely potent inflammatory peptide that exerts numerous effects on the vasculature, including vasodilation, and can increase vascular permeability; its binding to the bradykinin B2 receptor triggers an increase in intracellular Ca^2+^, endothelium-derived hyperpolarizing factor, prostacyclin, and nitric oxide production, eliciting relaxation of vascular smooth muscle cells and a drop in blood pressure [33, 34]. A direct relationship has been demonstrated between the kallikrein-kinin system and NHR [30]. In the present study, Kng1 was upregulated in the C4880 group, suggesting that C4880 may stimulate the coagulation pathway to generate F12, while F12 stimulates the production of Kng1 to activate the kallikrein-kinin system. Our results indicate that OVA could not activate the kallikrein-kinin system. Thus, Kng1 can be used as an important index for the kallikrein-kinin pathway.

Oxygen free radicals are generally related to various inflammatory diseases, and one study has indicated that oxygen free radicals are increased in Gpx1-deficient mice, suggesting that Gpx1 can protect the body from oxidative damage [35]. Gpx1 is also involved in the regulation of prostaglandin synthesis by generating 5-HETE from 5-HPETE, and these peroxidized phospholipids are reactive with glutathione peroxidase only after enzymatic attack by phospholipase A2 [36]. Gpx1 was downregulated in the C4880 group, suggesting that C4880 induced arachidonic acid release from mast cells through the activation of phospholipase A2, in accordance with a previous report [21]. These data suggest that Gpx1 may be an important protein in the direct stimulation pathway.

Seven proteins were selected according to the NHR generation pathways for further analysis by ELISA to confirm and supplement the proteomics data; the ELISA results were used in multiple linear regression analysis. The regression equation ${\hat{Y}}_{\text{symptom scores}}=-2.636+0.045X_{\text{Kng}1}+0.046X_{\text{F}13}-0.272X_{\text{Gpx}1}+0.012X_{\text{Sc}5\text{b}9}$ was obtained and the results show that Kng1, Gpx1, Sc5b9, and F13 were independently correlated with symptom scores (R = 0.488, p = 0.000; R = −0.422, p = 0.000; R = 0.301, p = 0.007; and R = 0.213, p = 0.049; respectively) (S4 Table), indicating that these proteins could reflect the presence of an NHR to some extent.

### Effect process

Histamine, as an early diagnostic marker of immediate phase hypersensitivity reactions, has been described as a "gold standard" of acute allergic monitoring and is a typical mediator that causes various pathophysiologic events in NHR [2, 37]. When the histamine level is: ≤ 1 ng·mL^−1^, there are no symptoms; 1–2 ng·mL^−1^, skin response only; 3 ng·mL^−1^, systemic reaction; > 100 ng·mL^−1^, serious reaction (allergic shock) [38]. The results of the present study show that the histamine level was higher in the C4880 group than in the OVA group. Thus, serious systemic reactions were caused by C4880 but mild symptoms such as trembling were caused by OVA, revealing that the concentration of histamine was intimately correlated with nonallergic symptoms in the animals.

Several other proteins including Prdx2, Cndp1, MYLPF, and DCD were also identified in this study. Prdx2 was significantly decreased and the others were upregulated in both groups. Prdx2 is a modulator of inflammatory and immune responses through its antioxidant activity [39], and is also a negative regulator of the pro-inflammatory Toll-like receptor 4 (TLR4) signaling pathway [40], which may act through TLR4 in NK cells, since these cells can produce type 1 chemokines and IFN-γ upon stimulation, resulting in enhancement of killing activity [41]. Thus, Prdx2 may be a protein that can be used to evaluating allergenicity [42]. In contrast, the reduction in Cndp1 might have an antagonistic effect on NHR in that the downregulated Cndp1 can maintain the activity of carnosine, which can inhibit inflammation- and fibrosis-related cytokines [43, 44]. Of interest, MYLPF has an important role in muscle contraction and was found to be expressed at lower levels in chronic heart failure patients, and oxidative stress due to hypoxia-reoxygenation could lead to protein modifications and its subsequent degradation [45]. One study has shown that the levels of MYLPF drastically decrease in cardiac myocytes during myocardial infarction; a decreased stroke volume during the entire reoxygenation period, which was due to deterioration of cardiac systolic function, was also observed [46], suggesting that MYLPF can be used to indicate shock or even death due to NHR. DCD, which is secreted by sweat glands, is an important component of the innate response in many species that may help limit infection by potential pathogens in the first few hours following bacterial colonization [47, 48]. In the present study, that DCD was downregulated may be because the temperature decreased the activity of sweat glands; hence, DCD can be used to indicate NHR trembling.

## Conclusion

To the best of our knowledge, this is the first study to analyze the mechanism of NHR through proteomics.

Various proteins such as prdx2, Cndp1, MYLPF, and DCD were found in this study and their potential utility as NHR-indicative potential biomarkers was demonstrated. And Gpx1, Sc5b9 (C4d and Bb), F13, Kng1, and IgE could be used as candidate biomarkers for the indication of the direct stimulation, complement (classical and alternative), coagulation, kallikrein-kinin, and integrated pathways respectively. Based on the findings in a model organism, and that observations in humans have not been explored which need further study. These results are different from those of previous studies in that they suggest that C4880 is not only involved in the direct stimulation pathway, but also activates the complement and kallikrein-kinin pathways through the coagulation pathway, and showed dose dependence. OVA causes NHR through co-activation of the coagulation, classical complement, and integrated pathways.

## Supporting Information

S1 Fig Determination of protein purity by SDS-PAGE. (TIF)

S2 Fig The generation process of nonallergic hypersensitivity reaction according to references and KEGG nomenclature using proteomics. (TIF)

S1 Table Proteins quantified before depleting the highly abundant proteins. (DOCX)

S2 Table Proteins quantified after depleting the highly abundant proteins. (DOCX)

S3 Table Identification and quantitative results of the 1987 proteins in this study. (XLSX)

S4 Table The multiple regression correlation analysis of several proteins with symptom scores. (XLSX)

## References

[1] Hanzlik PJ, Karsner HT. Anaphylactoid phenomena from the intravenous administration of various colloids, arsenicals and other agents. J Pharmacol Exp Ther. 1920;14: 379–423.

[2] Toman M, Krejci J, Pinka K, Mensik P. Causes of anaphylactoid reactions in cattle after administration of lipoid preparations. Vet Med (Praha). 1992;37: 417–426. 1481339

[3] Jurakic TR, Marinovic B, Lipozencic J. Nonallergic hypersensitivity to nonsteroidal antiinflammatory drugs, angiotensin-converting enzyme inhibitors, radiocontrast media, local anesthetics, volume substitutes and medications used in general anesthesia. Acta Dermatovenerol Croat. 2009;17: 54–69. 19386216

[4] Gomez E, Ariza A, Blanca-Lopez N, Torres MJ. Nonimmediate hypersensitivity reactions to iodinated contrast media. Curr Opin Allergy Clin Immunol. 2013;13: 345–353. 10.1097/ACI.0b013e328362b926 23743515

[5] Mi YN, Ping NN, Xiao X, Zhu YB, Liu J, Cao YX. The severe adverse reaction to vitamin K1 injection is anaphylactoid reaction but not anaphylaxis. PLoS One 2014;9: e90199 10.1371/journal.pone.0090199 24594861 PMC3942416

[6] Szebeni J. Complement activation-related pseudoallergy: a new class of drug-induced acute immune toxicity. Toxicology. 2005;216: 106–121. 16140450 10.1016/j.tox.2005.07.023

[7] Choi YH, Yan GH, Chai OH, Song CH. Inhibitory effects of curcumin on passive cutaneous anaphylactoid response and compound 48/80-induced mast cell activation. Anat Cell Biol. 2010;43: 36–43. 10.5115/acb.2010.43.1.36 21190003 PMC2998773

[8] Coors EA, Seybold H, Merk HF, Mahler V. Polysorbate 80 in medical products and nonimmunologic anaphylactoid reactions. Ann Allergy Asthma Immunol. 2005;95: 593–599. 16400901 10.1016/S1081-1206(10)61024-1

[9] Chanan-Khan A, Szebeni J, Savay S, Liebes L, Rafique NM, Alving CR, et al Complement activation following first exposure to pegylated liposomal doxorubicin (Doxil): possible role in hypersensitivity reactions. Ann Oncol. 2003;14: 1430–1437. 12954584 10.1093/annonc/mdg374

[10] Kobek M, Jankowski Z, Chowaniec C, Chowaniec M, Jablonski C, Skowronek R. Possibilities of post-mortem diagnostics, including immunodiagnostics, in cases of sudden death due to anaphylactic and anaphylactoid reactions. Arch Med Sadowej Kryminol. 2014;64: 102–111. 25574942 10.5114/amsik.2014.47745

[11] Yang R, Lao QC, Yu HP, Zhang Y, Liu HC, Luan L, et al Tween-80 and impurity induce anaphylactoid reaction in zebrafish. J Appl Toxicol. 2015;35: 295–301. 10.1002/jat.3069 25345596

[12] Xiang Z, Qiao T, Xiao H, Kang TG, Dou D, Li H, et al The anaphylactoid constituents in Xue-Sai-Tong injection. Planta Med. 2013;79: 1043–1050. 10.1055/s-0032-1328746 23839821

[13] Li Z, Gao Y, Wang H, Liu Z. A rat model of Shuang Huang Lian injection-induced anaphylaxis. Asian Pac J Allergy Immunol. 2010;28: 185–191. 21038789

[14] Li XM, Srivastava K, Grishin A, Huang CK, Schofield B, Burks W, et al Persistent protective effect of heat-killed Escherichia coli producing "engineered," recombinant peanut proteins in a murine model of peanut allergy. J Allergy Clin Immunol. 2003;112: 159–167. 12847493 10.1067/mai.2003.1622

[15] Nie JZ, An L, Miao K, Hou ZC, Yu Y, Tan K, et al Comparative analysis of dynamic proteomic profiles between in vivo and in vitro produced mouse embryos during postimplantation period. J Proteome Res. 2013;12: 3843–3856. 10.1021/pr301044b 23841881

[16] Xu CP, Li X, Hu YJ, Cui Z, Wang L, Liang L, et al Quantitative proteomics reveals ELP2 as a regulator to the inhibitory effect of TNF-α on osteoblast differentiation. J Proteomics. 2015;114: 234–246. 10.1016/j.jprot.2014.11.002 25486498

[17] Meng QG, Hou LB, Zhao Y, Huang X, Huang YQ, Xia SY, et al iTRAQ-based proteomic study of the effects of Spiroplasma eriocheiris on Chinese mitten crab Eriocheir sinensis hemocytes. Fish Shellfish Immun. 2014;40: 182–189. 10.1016/j.fsi.2014.06.029 25017370

[18] Mousli M, Bronner C, Landry Y, Bockaert J, Rouot B. Direct activation of GTP-binding regulatory proteins (G-proteins) by substance P and compound 48/80. FEBS Lett. 1990;259: 260–262. 1688415 10.1016/0014-5793(90)80023-c

[19] Palomaki VA, Laitinen JT. The basic secretagogue compound 48/80 activates G proteins indirectly via stimulation of phospholipase D-lysophosphatidic acid receptor axis and 5-HT1A receptors in rat brain sections. Br J Pharmacol. 2006;147: 596–606. 16415902 10.1038/sj.bjp.0706671 PMC1751339

[20] McNeil BD, Pundir P, Meeker S, Han L, Undem BJ, Kulka M, et al Identification of a mast-cell-specific receptor crucial for pseudo-allergic drug reactions. Nature 2015;519: 237–241. 10.1038/nature14022 25517090 PMC4359082

[21] Ferry X, Brehin S, Kamel R, Landry Y. G protein-dependent activation of mast cell by peptides and basic secretagogues. Peptides 2002;23: 1507–1515. 12182955 10.1016/s0196-9781(02)00090-6

[22] Asano E, Ebara T, Yamada-Namikawa C, Kitaori T, Suzumori N, Katano K, et al Genotyping analysis for the 46 C/T polymorphism of coagulation factor XII and the involvement of factor XII activity in patients with recurrent pregnancy loss. PLoS One 2014;9: e114452 10.1371/journal.pone.0114452 25489738 PMC4260909

[23] Ichinose A, McMullen BA, Fujikawa K, Davie EW. Amino acid sequence of the b subunit of human factor XIII, a protein composed of ten repetitive segments. Biochemistry-US. 1986;25: 4633–4638. 10.1021/bi00364a027 3021194

[24] Jakubowski HV, Kline MD, Owen WG. The effect of bovine thrombomodulin on the specificity of bovine thrombin. J Biol Chem. 1986;261: 3876–3882. 3005304

[25] Tarr J, Eggleton P. Immune function of C1q and its modulators CD91 and CD93. Crit Rev Immunol. 2005;25: 305–330. 16167883 10.1615/critrevimmunol.v25.i4.40

[26] Nauta AJ, Trouw LA, Daha MR, Tijsma O, Nieuwland R, Schwaeble WJ, et al Direct binding of C1q to apoptotic cells and cell blebs induces complement activation. Eur J Immunol. 2002;32: 1726–1736. 12115656 10.1002/1521-4141(200206)32:6<1726::AID-IMMU1726>3.0.CO;2-R

[27] Krishnan V, Xu Y, Macon K, Volanakis JE, Narayana SV. The structure of C2b, a fragment of complement component C2 produced during C3 convertase formation. Acta Crystallogr D Biol Crystallogr. 2009;65: 266–274. 10.1107/S0907444909000389 19237749 PMC2651757

[28] Aleshin AE, Schraufstatter IU, Stec B, Bankston LA, Liddington RC, DiScipio RG. Structure of complement C6 suggests a mechanism for initiation and unidirectional, sequential assembly of membrane attack complex (MAC). J Biol Chem. 2012;287: 10210–10222. 10.1074/jbc.M111.327809 22267737 PMC3323040

[29] Szebeni J, Muggia FM, Alving CR. Complement activation by Cremophor EL as a possible contributor to hypersensitivity to paclitaxel: an in vitro study. J Natl Cancer Inst. 1998;90: 300–306. 9486816 10.1093/jnci/90.4.300

[30] Gueant-Rodriguez RM, Romano A, Barbaud A, Brockow K, Gueant JL. Hypersensitivity reactions to iodinated contrast media. Curr Pharm Des. 2006;12: 3359–3372. 17017930 10.2174/138161206778193999

[31] Szebeni J, Baranyi L, Savay S, Milosevits J, Bodo M, Bunger R, et al The interaction of liposomes with the complement system: in vitro and in vivo assays. Methods Enzymol. 2003;373: 136–154. 14714402 10.1016/S0076-6879(03)73010-9

[32] Sala-Cunill A, Bjorkqvist J, Senter R, Guilarte M, Cardona V, Labrador M, et al Plasma contact system activation drives anaphylaxis in severe mast cell-mediated allergic reactions. J Allergy Clin Immunol. 2015;135: 1031–1043. 10.1016/j.jaci.2014.07.057 25240785

[33] Golias C, Charalabopoulos A, Stagikas D, Charalabopoulos K, Batistatou A. The kinin system—bradykinin: biological effects and clinical implications. Multiple role of the kinin system—bradykinin. Hippokratia. 2007;11: 124–128. 19582206 PMC2658795

[34] Giles TD, Sander GE, Nossaman BD, Kadowitz PJ. Impaired vasodilation in the pathogenesis of hypertension: focus on nitric oxide, endothelial-derived hyperpolarizing factors, and prostaglandins. J Clin Hypertens (Greenwich). 2012;14: 198–205. 22458740 10.1111/j.1751-7176.2012.00606.x PMC8108814

[35] Kim HR, Lee A, Choi EJ, Kie JH, Lim W, Lee HK, et al Attenuation of experimental colitis in glutathione peroxidase 1 and catalase double knockout mice through enhancing regulatory T cell function. PLoS One 2014;9: e95332 10.1371/journal.pone.0095332 24743300 PMC3990669

[36] Grossmann A, Wendel A. Non-reactivity of the selenoenzyme glutathione peroxidase with enzymatically hydroperoxidized phospholipids. Eur J Biochem. 1983;135: 549–552. 6413205 10.1111/j.1432-1033.1983.tb07687.x

[37] Hashimoto T, Ohata H, Honda K. Lysophosphatidic acid (LPA) induces plasma exudation and histamine release in mice via LPA receptors. J Pharmacol Sci. 2006;100: 82–87. 16404130 10.1254/jphs.fpj05030x

[38] Wang Z, Wang D, Sui Y, Cui H, Yu Y. Experimental study on anaphylaxis of qingkailing injection and its components on Beagle dogs. J Tradit Chin Med. 2012;32: 641–645. 23427403 10.1016/s0254-6272(13)60085-0

[39] Szabó KÉ, Line K, Eggleton P, Littlechild JA, Winyard PG. Structure and function of the human peroxiredoxin-based antioxidant system: the interplay between peroxiredoxins, thioredoxins, thioredoxin reductases, sulfiredoxins and sestrins In: Jacob C and Winyard PG, editors. Redox Signaling and Regulation in Biology and Medicine. Weinheim: Wiley-VCH Verlag GmbH & Co. KGaA; 2009 pp. 143–179.

[40] Yang CS, Lee DS, Song CH, An SJ, Li S, Kim JM, et al Roles of peroxiredoxin II in the regulation of proinflammatory responses to LPS and protection against endotoxin-induced lethal shock. J Exp Med. 2007;204: 583–594. 17325201 10.1084/jem.20061849 PMC2137909

[41] Ishii T, Warabi E, Yanagawa T. Novel roles of peroxiredoxins in inflammation, cancer and innate immunity. J Clin Biochem Nutr. 2012;50: 91–105. 10.3164/jcbn.11-109 22448089 PMC3303482

[42] Hobson DJ, Rupa P, Diaz GJ, Zhang H, Yang M, Mine Y, et al Proteomic analysis of ovomucoid hypersensitivity in mice by two-dimensional difference gel electrophoresis (2D-DIGE). Food Chem Toxicol. 2007;45: 2372–2380. 17897766 10.1016/j.fct.2007.06.039 PMC7126535

[43] Gautam P, Nair SC, Gupta MK, Sharma R, Polisetty RV, Uppin MS, et al Proteins with altered levels in plasma from glioblastoma patients as revealed by iTRAQ-based quantitative proteomic analysis. PLoS One 2012;7: e46153 10.1371/journal.pone.0046153 23029420 PMC3461020

[44] Kilis-Pstrusinska K. Carnosine, carnosinase and kidney diseases. Postepy Hig Med Dosw (Online). 2012;66: 215–221. 22706107 10.5604/17322693.991600

[45] Li Y, Wu G, Tang Q, Huang C, Jiang H, Shi L, et al Slow cardiac myosin regulatory light chain 2 (MYL2) was down-expressed in chronic heart failure patients. Clin Cardiol. 2011;34: 30–34. 10.1002/clc.20832 21259275 PMC6652384

[46] Doroszko A, Polewicz D, Cadete VJ, Sawicka J, Jones M, Szczesna-Cordary D, et al Neonatal asphyxia induces the nitration of cardiac myosin light chain 2 that is associated with cardiac systolic dysfunction. Shock 2010;34: 592–600. 10.1097/SHK.0b013e3181e14f1d 20386496 PMC3084583

[47] Schittek B, Hipfel R, Sauer B, Bauer J, Kalbacher H, Stevanovic S, et al Dermcidin: a novel human antibiotic peptide secreted by sweat glands. Nat Immunol. 2001;2: 1133–1137. 11694882 10.1038/ni732

[48] Rieg S, Garbe C, Sauer B, Kalbacher H, Schittek B. Dermcidin is constitutively produced by eccrine sweat glands and is not induced in epidermal cells under inflammatory skin conditions. Br J Dermatol. 2004;151: 534–539. 15377337 10.1111/j.1365-2133.2004.06081.x

