# Supplementary figures and images for: Proteomics Study on Nonallergic Hypersensitivity Induced by Compound 4880 and Ovalbumin
Source: PLoS One. 2016 Feb 1;11(2):e0148262. doi: 10.1371/journal.pone.0148262 (PMC4734762; doi:10.1371/journal.pone.0148262)

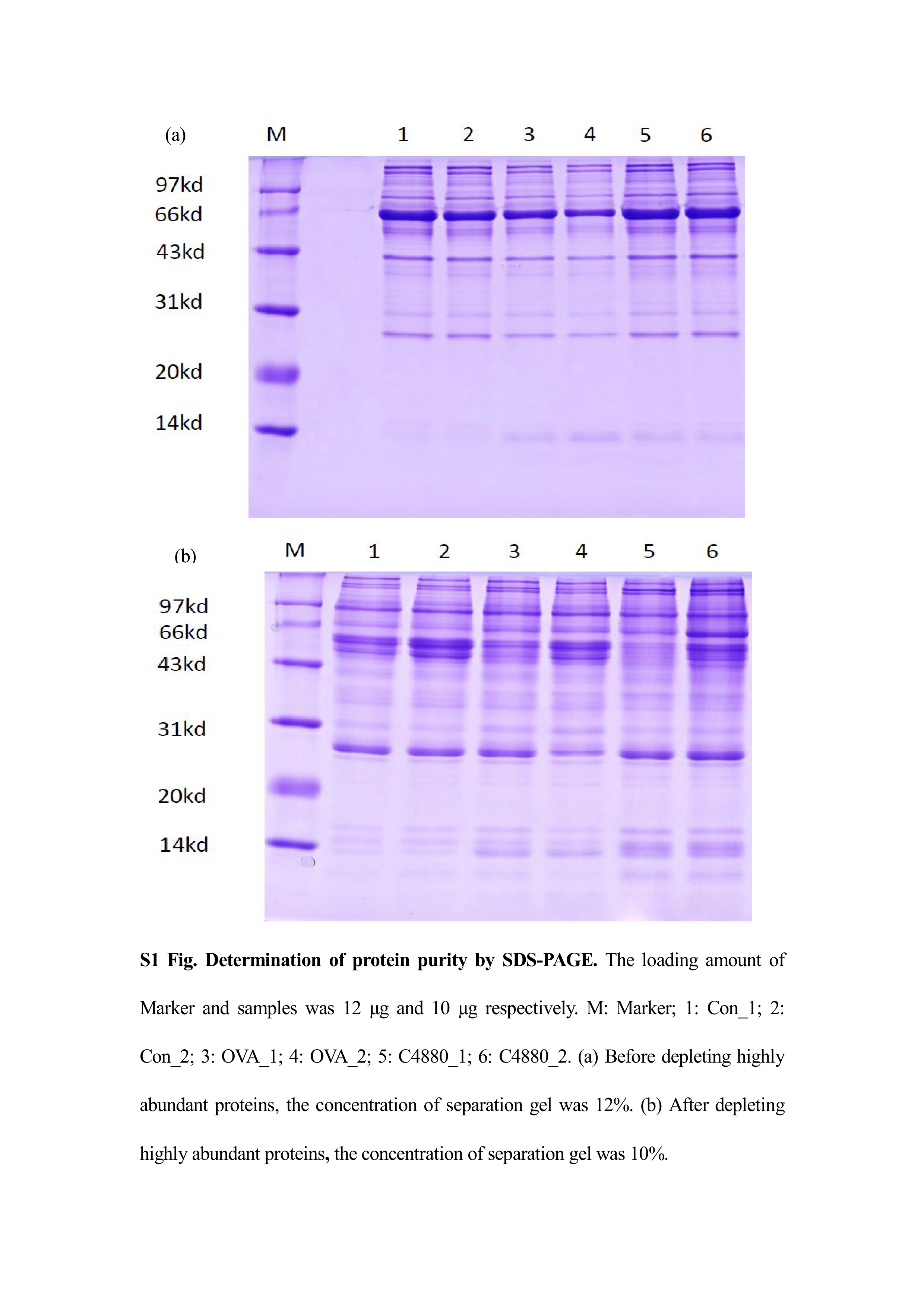

Supplement: S1 Fig — (TIF) [file pone.0148262.s001.tif]

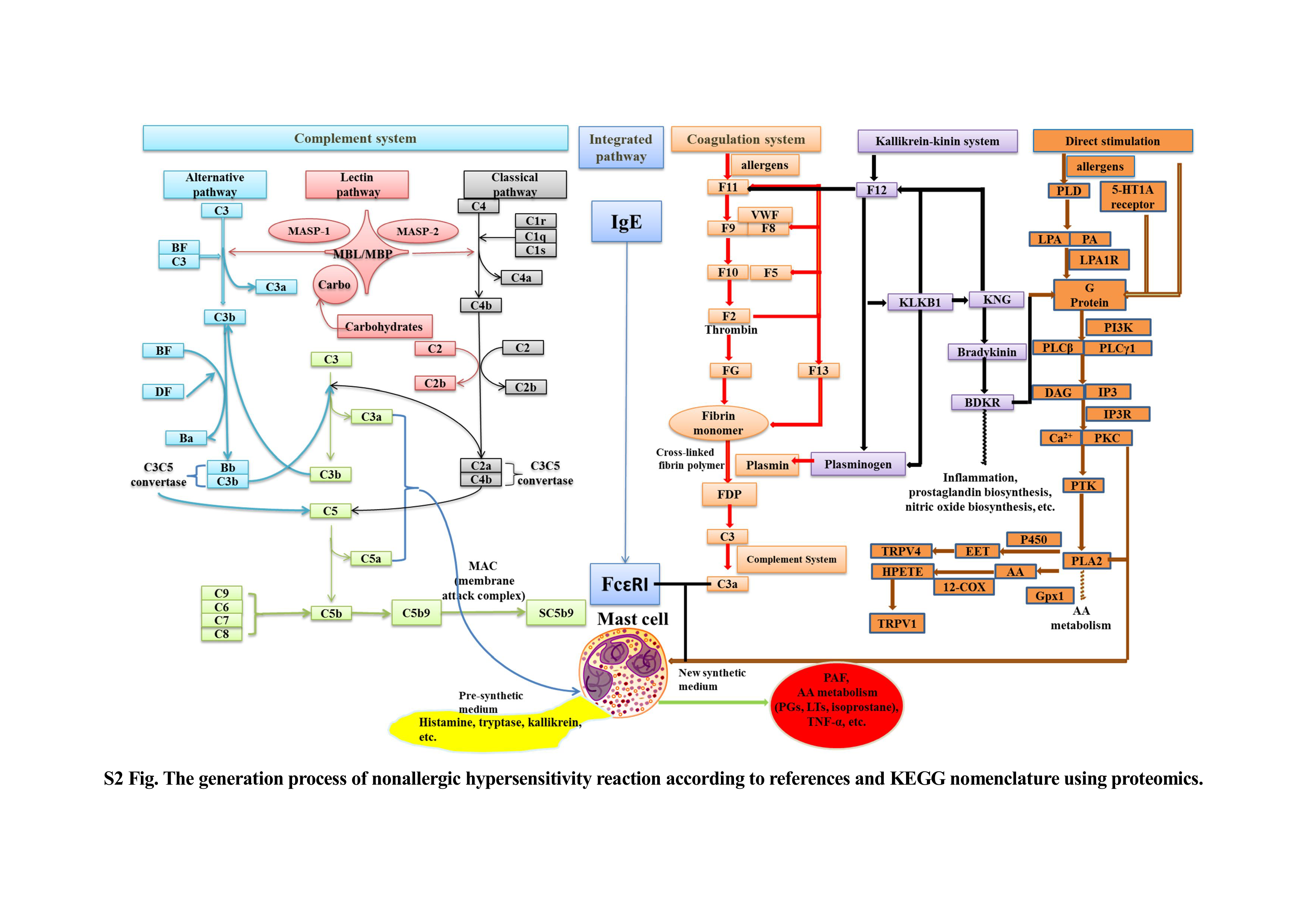

Supplement: S2 Fig — (TIF) [file pone.0148262.s002.tif]
