# Supplementary material for: Proteomics Study on Nonallergic Hypersensitivity Induced by Compound 4880 and Ovalbumin
Source: PLoS One. 2016 Feb 1;11(2):e0148262. doi: 10.1371/journal.pone.0148262 (PMC4734762; doi:10.1371/journal.pone.0148262)
Supplement: S1 Table — (DOCX) [file pone.0148262.s003.docx]

**Table S1. Proteins quantified before depleting the highly abundant proteins**

| ID | Concentration (μg/μL) | Volume (μL) | Total protein (μg) |
| --- | --- | --- | --- |
| Con_1 | 56.75 | 1000 | 56755.08 |
| Con_2 | 55.48 | 1000 | 55484.30 |
| OVA_1 | 43.01 | 1000 | 43007.60 |
| OVA_2 | 47.40 | 1000 | 47397.55 |
| C4880_1 | 47.28 | 1000 | 47282.03 |
| C4880_2 | 52.02 | 1000 | 52018.55 |
