# Supplementary material for: Proteomics Study on Nonallergic Hypersensitivity Induced by Compound 4880 and Ovalbumin
Source: PLoS One. 2016 Feb 1;11(2):e0148262. doi: 10.1371/journal.pone.0148262 (PMC4734762; doi:10.1371/journal.pone.0148262)
Supplement: S2 Table — (DOCX) [file pone.0148262.s004.docx]

**Table S2. Proteins quantified after depleting the highly abundant proteins**

| ID | Concentration (μg/μL) | Volume (μL) | Total protein (μg) |
| --- | --- | --- | --- |
| Con_1 | 1.09 | 200 | 218.37 |
| Con_2 | 1.18 | 200 | 235.01 |
| OVA_1 | 0.93 | 200 | 185.10 |
| OVA_2 | 1.27 | 200 | 253.49 |
| C4880_1 | 1.22 | 200 | 244.25 |
| C4880_2 | 1.68 | 200 | 336.67 |
